# Deciphering the killing mechanisms of potassium iodide in combination with antimicrobial photodynamic therapy against cross-kingdom biofilm

**DOI:** 10.3389/fcimb.2024.1444764

**Published:** 2024-10-15

**Authors:** Yijun Li, Shan Huang, Jingyun Du, Shaofeng Wang, Zhiyu Cai, Xiaojing Huang

**Affiliations:** ^1^ Fujian Key Laboratory of Oral Diseases and Fujian Provincial Engineering Research Center of Oral Biomaterial and Stomatological Key Lab of Fujian College and University, School and Hospital of Stomatology, Fujian Medical University, Fuzhou, China; ^2^ Stomatological Hospital of Xiamen Medical College and Xiamen Key Laboratory of Stomatological Disease Diagnosis and Treatment, Xiamen, China; ^3^ Department of Stomatology, Zhongshan Hospital Affiliated to Xiamen University, Xiamen, China; ^4^ Department of Stomatology, Fujian Medical University Union Hospital, Fuzhou, China

**Keywords:** antimicrobial photodynamic therapy, potassium iodide, *Streptococcus mutans*, *Candida albicans*, cross-kingdom biofilm, antimicrobial mechanism

## Abstract

**Introduction:**

The co-existence of *S. mutans* and *C. albicans* is frequently detected in root caries and early child caries and is reported to be associated with recurrent caries. The aim of this study was to investigate the effects of potassium iodide (KI) in combination with toluidine blue O-mediated antimicrobial photodynamic therapy (aPDT) on *S. mutans* and *C. albicans* mixed-species biofilm, as well as the antibiofilm mechanisms involved.

**Methods:**

Mixed-species biofilm was constructed of *S. mutans* and *C. albicans* on dentin blocks. The antibiofilm efficacy, cytotoxicity and antibiofilm mechanism of KI in combination with aPDT were determined and evaluated.

**Results:**

KI+TBO-aPDT treatment caused reduction in microorganism counts, metabolic activity, and biofilm biomass of mixed-species biofilm without inducing cytotoxicity to hDPCs (human dental pulp cells). Observations such increased ROS (reactive oxygen species) levels, impaired cell membrane function, cell apoptosis and reduced expression in several genes seem to be artifacts of reduced growth and general killing by KI+TBO-aPDT treatment.

**Discussion:**

These data suggested that KI in combination with aPDT as an innovative approach to combat *S. mutans* and *C. albicans* biofilm, and thus as an optional treatment for caries.

## 1 Introduction

Within the oral microbiome, *Streptococcus mutans* is regarded as a prominent carious pathogen that adheres tightly to the tooth surface and rapidly initiates the formation of carious biofilm (Shanmugam et al., 2020). However, the carious lesions are not an outcome of *S. mutans* solely, but rather a result of mutual activities of polymicrobial biofilm. Recent clinical evidence has revealed the co-existence of *Candida albicans* and *S. mutans* in carious lesions such as early childhood caries, root caries, and even recurrent caries (de Carvalho et al., 2006; Xiao et al., 2018; Du et al., 2020; Garcia et al., 2021). To further understand the intricate interaction between *C. albicans* and *S. mutans*, consequent studies have used *in vitro* and *in vivo* models to shed light on the interspecies interactions. These findings suggested that the co-existence of *C. albicans* and *S. mutans* could enhance co-species biofilm matrix production, modulate the virulence factor expression, and finally contribute to cross-kingdom biofilm formation (He et al., 2017; Lobo et al., 2019). The presence of extracellular polymeric substance (EPS) matrix formed by cross-kingdom biofilm with its altered microenvironment confines drug entry and triggers antimicrobial tolerance, making this co-species biofilm challenging to treat (Kim et al., 2018).

Treatment regimens ranging from natural compounds to antimicrobial peptides or nanoparticles have made significant progress in tackling *C. albicans* and *S. mutans* biofilm (Priya et al., 2021; Ito et al., 2022; Zeng et al., 2022; Li et al., 2023). Nevertheless, these treatment modalities have certain limitations that constrain their widespread application, such as limited antimicrobial capacity, potential antimicrobial resistance, and high manufacturing cost.

Antimicrobial photodynamic therapy (aPDT), as a non-antibiotic approach, has garnered considerable interest for its conservative, selective, and safe properties (Cieplik et al., 2018). It involves the photochemical reactions of photosensitizers and the generation of reactive oxygen species (ROS) under light irradiation. While the advantages of aPDT in terms of biofilm elimination have been well-demonstrated (Balhaddad et al., 2021), its limited effectiveness on complex carious biofilms has been observed in clinical settings (Quishida et al., 2015). The reasons behind the limited antimicrobial capacity of aPDT on caries biofilm are due to several factors, such as short lifespan and limited diffusion distance of ROS, insufficient penetration of photosensitizers into abundant biofilm matrix, and the complicated structure of dentin tubules (Balhaddad et al., 2020). Therefore, endeavors from laboratories have been made to explore different ways to improve the antimicrobial effectiveness of aPDT. In 2015, Vecchio et al. discovered that combining the photosensitizer with potassium iodide (KI) could enhance killing against *Staphylococcus aureus* (*S. aureus*) and *Escherichia coli* (*E. coli*) (Vecchio et al., 2015). The addition of KI into aPDT procedure reduces the concentration of photosensitizer and light dose, thus minimizing potential side effects. Our previous work also revealed that toluidine blue O (TBO)-mediated aPDT (TBO-aPDT) plus KI could result in enhanced killing in *C. albicans* and *S. mutans* these two microbes (Li et al., 2022). Meanwhile, the mechanism by which KI in combination with TBO-aPDT can exert an antimicrobial effect probably relies on the generation of iodide radical species and hydrogen peroxide (H_2_O_2_), which, in synergy, could inflict more severe damage on microbial cells.

While some studies have partially examined the antimicrobial effect of KI in combination with aPDT on caries biofilms, they often lack cytotoxicity evaluation, comparison with current antimicrobials, and models that closely resemble the clinical situation. Furthermore, the mechanisms underlying the antimicrobial and anti-biofilm effects of this combination have not been fully understood. The aim of this study was to investigate the effects of TBO-aPDT plus KI on *C. albicans* and *S. mutans* mixed-species biofilm, as well as the antibiofilm mechanisms involved.

## 2 Materials and method

### 2.1 Microorganism culture and growth condition

The microorganism strains used in this study were *C. albicans* ATCC 10231 and *S. mutans* UA159. *C. albicans* was cultivated aerobically on sabouraud dextrose agar (SDA) plate at 37°C, whilst *S. mutans* was maintained on brain heart infusion (BHI) agar plate anaerobically at 37°C. For inoculum preparation, *C. albicans* and *S. mutans* cells were grown on sabouraud dextrose broth (SDB) and BHI broth respectively to mid-exponential phase.

### 2.2 *In vitro* mixed-species biofilm formation

After reaching mid-exponential phase, bacterial cells and fungal cells were collected by centrifugation (4000 rpm, 10 min) respectively and the resulting pellet was washed three times in sterile phosphate-buffered saline (PBS). *C. albicans* suspension was adjusted to 1×10^7^ CFU/mL and *S. mutans* was adjusted to 1×10^8^ CFU/mL for inoculation. Sterilized bovine dentin blocks (7×1.4×1.2 mm) were used as the surface of mix-species biofilm formation.

Before mix-species biofilm formation, the bovine dentin disks were coated with 1 mL artificial saliva in 24-well plate for 1 h at 37°C. After formation of the salivary pellicle, 100 μL of each microbe suspension plus 1 mL tryptone-yeast extract (TYE) broth was incubated simultaneously on saliva-coated bovine dentin blocks which were positioned vertically in 24-well plate. The biofilms were grown at 37°C in liquid culture medium TYE broth supplemented with 1% sucrose under anaerobic conditions for 48 h. The culture medium was refreshed every 24 h.

### 2.3 Treatment protocols

After inoculation with mixed-species biofilm, the dentin blocks were rinsed with PBS to remove loosely attached microorganisms and subjected to different treatments. The dentin blocks were randomly divided into six groups and the details of experimental groups were shown in **Table 1**.

**Table 1:** Experimental group.

Group	TBO (μg/mL)	KI (mM)	Time of Treatment
PBS(control)	0	0	No treatment
TBO-aPDT-L	1	0	60 s
KI+TBO-aPDT-L	1	200	60 s
TBO-aPDT-H	20	0	120 s
KI+TBO-aPDT-H	20	200	120 s
0.2% CHX	0	0	120 s

1. Control group-Dentin blocks were rinsed with PBS and not received any further treatment.
2. aPDT in low dosage group (TBO-aPDT-L group) -TBO (Sigma-Aldrich, S. Louis, MO, USA) was used as a photosensitizer and was prepared in PBS. Dentin blocks were immersed in 1 μg/mL TBO solution, incubated at dark for 30 min and then irradiated with red light. The light source was a light-emitting diode (LED; Denfotex, Redhill, UK) which was centered at 635 nm with a bandwidth of 10 nm. The radiation distance between the light to the dentin block surface was set at 1 cm. The output power was 530 mW and the beam diameter was 1.3 cm. Power measurements were measured with a power meter (Ophir Optronics Ltd., Danvers, MA, USA). The irradiation time was set as 60s and the applied output energy density was 24 J/cm^2^.
3. KI plus aPDT in low dosage (KI+TBO-aPDT-L group) - KI (Sigma-Aldrich, St. Louis, MO, USA) was dissolved in sterilized distilled water. Dentin blocks were incubated with 1 μg/mL TBO and 200 mM KI solution for 30 min. Then dentin blocks were exposed to light source for 60 s.
4. aPDT in high dosage group (TBO-aPDT-H group) - Dentin blocks were immersed in 20 μg/mL TBO solution, incubated at dark for 30 min and then irradiated with red light.
5. KI plus aPDT in high dosage (KI+TBO-aPDT-H group) - Dentin blocks were incubated with 20 μg/mL TBO and 200 mM KI then exposed to light source for 120 s.
6. 0.2% (v/v) Chlorhexidine (CHX) group- Dentin blocks were immersed in CHX solution for 120 s and were gently rinsed with PBS solution to remove excess CHX.

### 2.4 Effect of TBO-aPDT plus KI on biofilm CFU counts

After treatments, the dentin block was washed gently by PBS to remove non-adherent microbes. Then the biofilm on dentin block surface was collected by sterile swabs (Wu et al., 2021). The biofilm was harvested by sonication and vortexing, serially diluted, and plated on agar plates. The agar plates were incubated at 37 °C for 48 h for counting. For species isolation, mitis salivarius agar (MSA; Hopebio, Qingdao, China) supplemented with 0.2 U/mL bacitracin (Hopebio, Qingdao, China) was used for *S. mutans* counting and Chromogenic Candida Agar was used for *C. albicans* counting.

### 2.5 Effect of TBO-aPDT plus KI on biofilm metabolic activity

3-(4,5-dimethylthiazol-2-yl)-2,5-diphenyl tetrazolium bromide (MTT) assay was performed to measure the metabolic activity of biofilms after treatments. MTT dye was dissolved in PBS and prepared at a final working concentration of 0.5 mg/mL. The blocks were rinsed gently with PBS and the dentin block was put on the bottom of a new 24-well plate. 1 mL TYE broth containing MTT was added to each well for 4 h at 37°C in 5% CO_2_. Then the medium was removed and 1 mL dimethyl sulfoxide (DMSO) was used to solubilize the formazan crystal for 20 min at 37°C. The absorbance of each well was determined at 492 nm via a microplate reader (SpectraMax iD3, Molecular Devices, Sunnyvale, CA, USA).

### 2.6 Effect of TBO-aPDT plus KI on biofilm formation

Crystal violet staining was used to quantify biofilm biomass of dentin block. The treated dentin blocks were gently washed with PBS and fixed in methanol for 15 min on a new 48-well plate. Then each dentin block was stained with 400 μL of 1% crystal violet for 15 min and washed with PBS to remove excess dye. For quantitative measurement of the biofilm biomass, the bounded crystal violet stain was dissolved completely in 100% ethanol for 1 h. Aliquots of 200 μL of the supernatant was transferred to a new 96-well plate and OD_590 nm_ absorbance was measured.

### 2.7 Effect of TBO-aPDT plus KI on biofilm structure

Three samples were randomly selected from each experimental group to observe biofilm structure and dentin surface change. The treated samples were fixed with 2.5% glutaraldehyde overnight, dehydrated in serial concentrations of ethanol, and eventually dried at room temperature. The samples were sputter coated with gold and observed under scanning electron microscope (SEM; FEI, Hillsboro, OR, USA). Photographs were taken to observe the biofilm structure and remaining microorganisms.

### 2.8 Effect of TBO-aPDT plus KI on live/dead microorganisms ratio

Three specimens per group were stained with the BacLight live/dead bacterial viability kit (Molecular Probes, Eugene, OR, USA) and observed under a fluorescence microscope. Each specimen was taken in five randomly selected fields of view. Live bacteria were stained with SYTO 9 emitting green fluorescence while the compromised microorganisms were stained with propidium iodide (PI) emitting red fluorescence. The stained samples were also observed using an Axio Observer 3 microscope (Carl Zeiss, Oberkochen, Germany) under a 20 × air objective with a magnification of 200 ×.

### 2.9 *In vitro* cytotoxicity assessment

#### 2.9.1 Human dental pulp stem cells culture

This study was reviewed and approved by the Ethics Committee of the stomatological hospital of Fujian Medical University (2020-41). Human dental pulp was isolated and cultured as described previously (Lan et al., 2022). Briefly, the isolated cells were cultured in α-minimum essential medium (α-MEM; Hyclone, Logan, Utah, USA) supplemented with 10% fetal bovine serum (FBS; Gibco, CA, USA) and 1% penicillin/streptomycin (Solarbio, Beijing, China) at 37°C in a humidified atmosphere of 5% CO_2_.

#### 2.9.2 Treatment protocol

To mimic the clinical situation, we seeded hDPCs on dentin block (7*6*1.2 mm) by using an *in vitro* pulp chamber model (Wang et al., 2023) (**Supplementary Figure S1**). hDPCs from the 3rd to 5th passage were seeded on the pulpal side of the dentin block (6×10^4^ cells/each dentin block) and then cultured in the model for 24 hours. After 24 hours of culture, hDPCs reached approximately 70% confluence and were ready for treatment. The experimental groups were shown in **Table 2**.

**Table 2:** Experimental group.

Group	TBO (μg/mL)	KI (mM)	Time of Treatment
Control	0	0	No treatment
KI+TBO-aPDT-L	1	200	60 s
KI+TBO-aPDT-H	20	200	120 s

#### 2.9.3 Cell counting kit-8 assay

After 24 h of additional incubation, hDPCs proliferation on the treated dentin block was evaluated using CCK-8 assay. Briefly, hDPCs on dentine blocks were washed with 200 μL of DMEM, incubated in a mixture of 90 μL of DMEM and 10 μL of CCK-8 reagent for 2 h at 37°C and 5% CO_2_. After incubation, the reaction mixture was transferred into a new 96-well plate. The optical density of each well was determined at 450 nm using a microplate reader.

#### 2.9.4 SEM observation of cell attachment

After treatments, samples (n=3/group) were fixed in 2.5% glutaraldehyde at 4°C overnight. Each dentin block was then washed twice in PBS and dehydrated through serial concentrations of ethanol. After that, the samples were washed three times with tert-butanol, dried by lyophilization and sputter coated with gold. Finally, the hPDCs were observed under the SEM at ×2000 magnification.

### 2.10 Exploring the antimicrobial mechanisms of TBO-aPDT plus on biofilm

#### 2.10.1 Transmission electron microscopy observation

For TEM observation, the treated biofilms on dentin block surface were collected by swab and then vortexed in PBS for 1min to disperse. The biofilm cells were prefixed in 1.5% paraformaldehyde, 2.5% glutaraldehyde fixative for 2 hours. Then the samples were prefixed in 2% osmium tetroxide and dehydrated with graded ethanol. After dehydration, the samples were incubated twice in acetone for 20 min and incubated overnight in a mixture of Araldite epoxy resin and acetone (1:1). Subsequently, the samples were transferred to a 2% Araldite mixture overnight, placed into silicone molds and polymerized for 48 h at 65°C. Ultrathin sections of the samples were obtained with a diamond knife (80-100 nm). Finally, the samples were viewed, and characteristic micrographs were taken with a TEM (Hitachi H7650, Tokyo, Japan) at magnifications varying from 2000 to 12000 folds.

#### 2.10.2 ROS probe study

Endogenous ROS production in microbial cells after treatment was determined by using 2′, 7′- dichlorofluorescein-diacetate (DCFHA; MKbio, Shanghai, China) probe. The collected biofilm cells from each experimental group were centrifuged, washed with PBS three times, and resuspended in 1 mL PBS. Then the samples were incubated with 10 μM DCFH-DA for 30 min. DCFH-DA could easily penetrate into cell membrane and is deacetylated by esterases to form non-fluorescent DCFH. DCFH would react with intracellular ROS to form fluorescent product 2,7-dichlorofluorescein (DCF). The fluorescence intensities of the samples were measured with a microplate reader (excitation, 485 nm, emission, 535 nm) after 1 h under shaking at 37°C. The fluorescence intensities values were normalized to the amount of viable cells in biofilms assessed by CFU assay describe above.

#### 2.10.3 Flow cytometry analysis

SYTOX green and DiBAC_4_(3) were used as fluorescent dyes to evaluate cell membrane permeability and cell membrane potential respectively by BD Accuri C6 flow cytometry machine (BD Falcon; San Jose, CA, USA). Biofilms were constructed, treated and brought to suspension as described above. For cell membrane permeability detection, the samples were incubated with SYTOX green at a final concentration of 1 μM and incubated for 15 min at room temperature in the dark (Panteleev et al., 2018). The percentage of microbial cells with compromised membrane in the suspension was determined by flow cytometry. The excitation wavelength is 480 nm and the emission wavelength is 530 nm. As for plasma membrane potential measurement, DiBAC_4_(3) was added into the samples at a final concentration of 0.5 μg/mL in PBS and then incubated for 15 min at room temperature in the dark. The percentage of depolarized fluorescent microbial cells in the suspension was determined by flow cytometry. The excitation wavelength is 490 nm and the emission wavelength is 525 nm. At least 10,000 events were tested for each sample.

For apoptotic analysis, the treated samples were stained with Annexin V-FITC apoptosis kit (Beyotime, Jiangsu, China). Biofilms were constructed, treated and brought to suspension as described above. Briefly, 195 μL binding buffer was added to each sample in accordance with manufacture protocols. Then, 5 μL of Annexin V-FITC and 10 μL of PI were added, and the cells were gently vortexed. Cells were incubated for 15 min at room temperature in the dark. At least 10,000 events were tested for each sample. Each sample was analyzed by flow cytometry using 488 nm for excitation and 530 nm for emission. Data were collected from at least 10,000 cells. Data were analyzed by Flowjo 6.2.1.

#### 2.10.4 RT-PCR analysis

In brief, the treated biofilm samples were collected from dentin block surface, treated with RNA protect reagent to stabilize RNA, and stored at −80°C. Total RNA was extracted from mixed biofilms according to the manufacturer’s instructions. RNA concentration and purity were determined spectrophotometrically using a Nanodrop (Wilmington, DE, USA). Expression of *S. mutans* biofilm, adhesin, and oxidative tolerance genes (*gtfB, gtfC, gtfD, ftf, gbpA, gbpB, aphcF, dpr*) and *C. albicans* hyphal/yeast-specific, adhesion and oxidative tolerance genes, as well as cell membrane synthesis genes (*als1, als3, bcr1, hwp1, ece1, cat1, sod1, trx1, erg11*) were analyzed. cDNA was reverse transcribed and used as the template. The quantitative polymerase chain reaction (qPCR) reactions were performed in LightCycler^®^ 480 (Roche, Basel, Switzerland) using 2× SYBR Green PCR Master Mix (Takara, Tokyo, Japan) and gene-specific primers listed in **Supplementary Tables S1**, **S2** to analyze the expression level of related genes. The PCR involved denaturation at 95 °C for 10 min, followed by 40 cycles of amplification (95 °C for 10 s, 55 °C for 10 s, and 72 °C for 10 s) and quantification. Different gene expression levels were normalized to 16S rRNA gene levels. Data were analyzed according to 2^−ΔΔCT^ method.

### 2.11 Statistical analysis

All statistical analysis were performed in SPSS software, version 16.0 (SPSS Inc., Chicago, IL, USA). The Shapiro–Wilk test for normality and Levene’s variance homogeneity test were applied to the data. Statistical analysis was performed by one-way analysis of variance followed by Tukey HSD test. The significance level α was set at 0.05.

## 3 Result

### 3.1 TBO-aPDT plus KI effectively inhibit mix-species biofilm viability, metabolic activity and biomass formation

**Figure 1:**
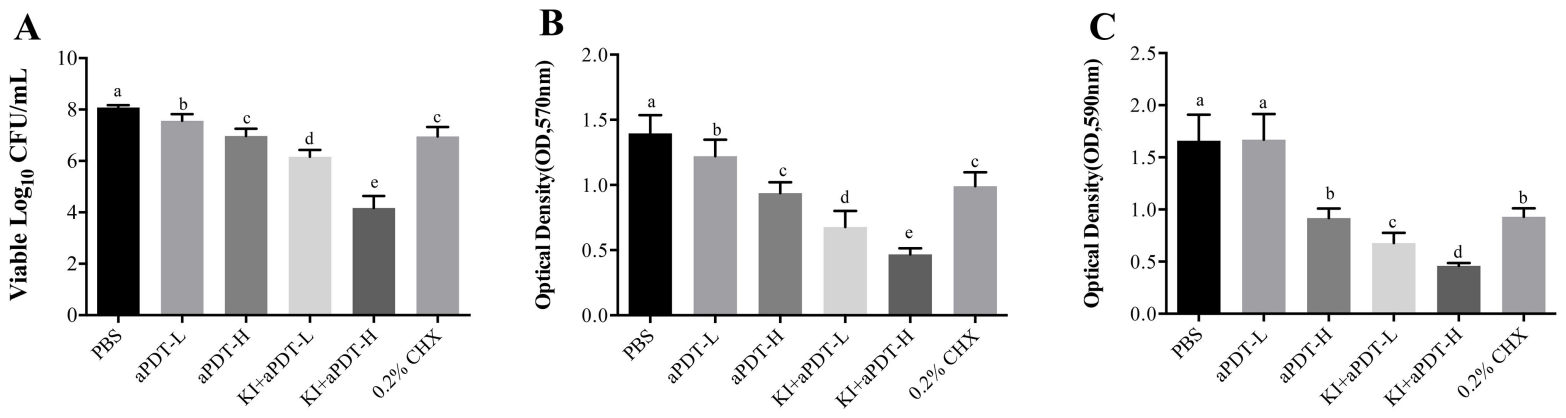
**(A)** The effect of different treatments on microbe numbers on dentin block surface detected by CFU counting (mean ± SD, n=6/group). **(B)** The effect of different treatments on biofilm metabolic activity on dentin block surface detected by MTT assay (mean ± SD, n=6/group). **(C)** The effect of different treatments on biofilm biomass on dentin block surface detected by crystal violet staining (mean ± SD, n=6/group). Values with dissimilar letters indicated significant difference between two groups (*P*<0.05).

Compared to control group, all treatment protocols could significantly decrease viable microbial cells of dentin block surface (**Figure 1A**). Among all experiment groups, TBO-aPDT-L treatment eliminated the fewest microorganisms from the dentin block surface (*P*<0.05). There is no significant difference regarding viable CFU counts between TBO-aPDT-H group and CHX group (*P*>0.05). When compared to CHX group or aPDT group, KI+TBO-aPDT could yield more reduction in viable microbe counts (**Figure 1A**). The most dramatic killing equals to 3 logs was observed when 20 μg/mL TBO plus KI were photoactivated.

In comparison with PBS group, all experimental groups could significantly reduce metabolic activity of biofilms (**Figure 1B**). The decrease trend is various among groups. When compared to CHX group or aPDT group, KI+TBO-aPDT groups had lower MTT values (*P*<0.05), indicating lower metabolic activity. The lowest value of metabolic activity was observed in KI+TBO-aPDT-H group (*P*<0.05).

All treatment protocols except TBO-aPDT-L group reduced more biofilm biomass of dentin block surface when compared to PBS group (**Figure 1C**). When compared to CHX group, there is no significant difference between CHX group and TBO-aPDT-H group (*P*>0.05). Using KI in association with TBO-aPDT could decrease biofilm biomass significantly than aPDT group or CHX group (**Figure 1C**).

### 3.2 TBO-aPDT plus KI destroy mix-species biofilm structure and modify live/dead ratio in mix-species biofilm

**Figure 2:**
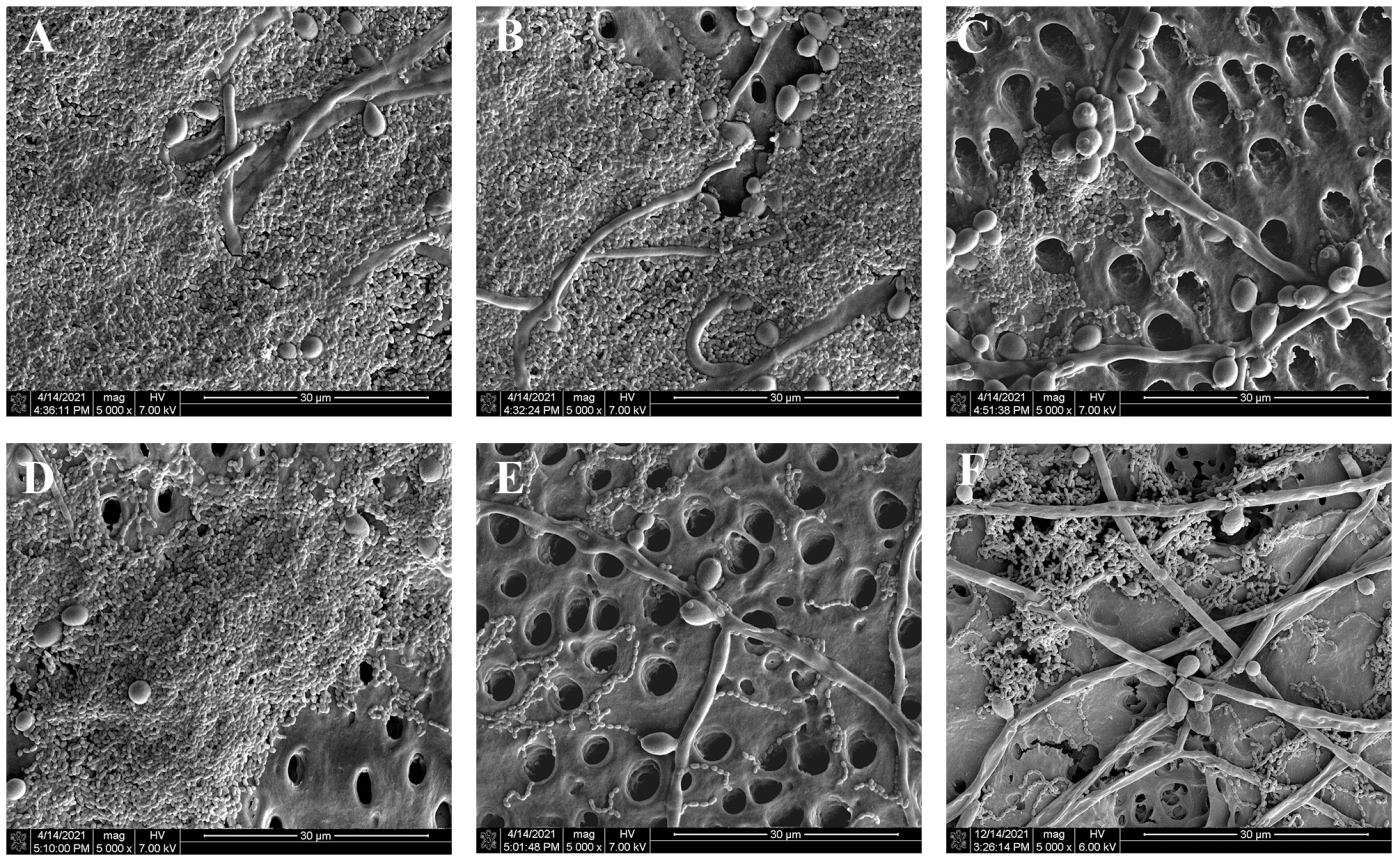
SEM observation of the effects of different treatments on the biofilm structure and morphology of microbes on the surface of dentin blocks, **(A)** PBS group, **(B)** TBO-aPDT-L group, **(C)** KI+TBO-aPDT-L group, **(D)** TBO-aPDT-H group, **(E)** KI+TBO-aPDT-H group, **(F)** 0.2% CHX group.

There are substantial numbers of *S. mutans* and *C. albicans* forming thick biofilms on the surface of dentin block in PBS group (**Figure 2A**). Compared to PBS group, the counts of two microorganisms became less and the opening of dentin tubule increased more in other treatment groups (**Figures 2B–F**). KI+TBO-aPDT-H group presented the fewest remaining microorganisms and most exposure of dentin tubule on the surface (**Figure 2F**). However, the cell morphology of *S. mutans* and *C. albicans* did not change after all treatment protocols.

**Figure 3:**
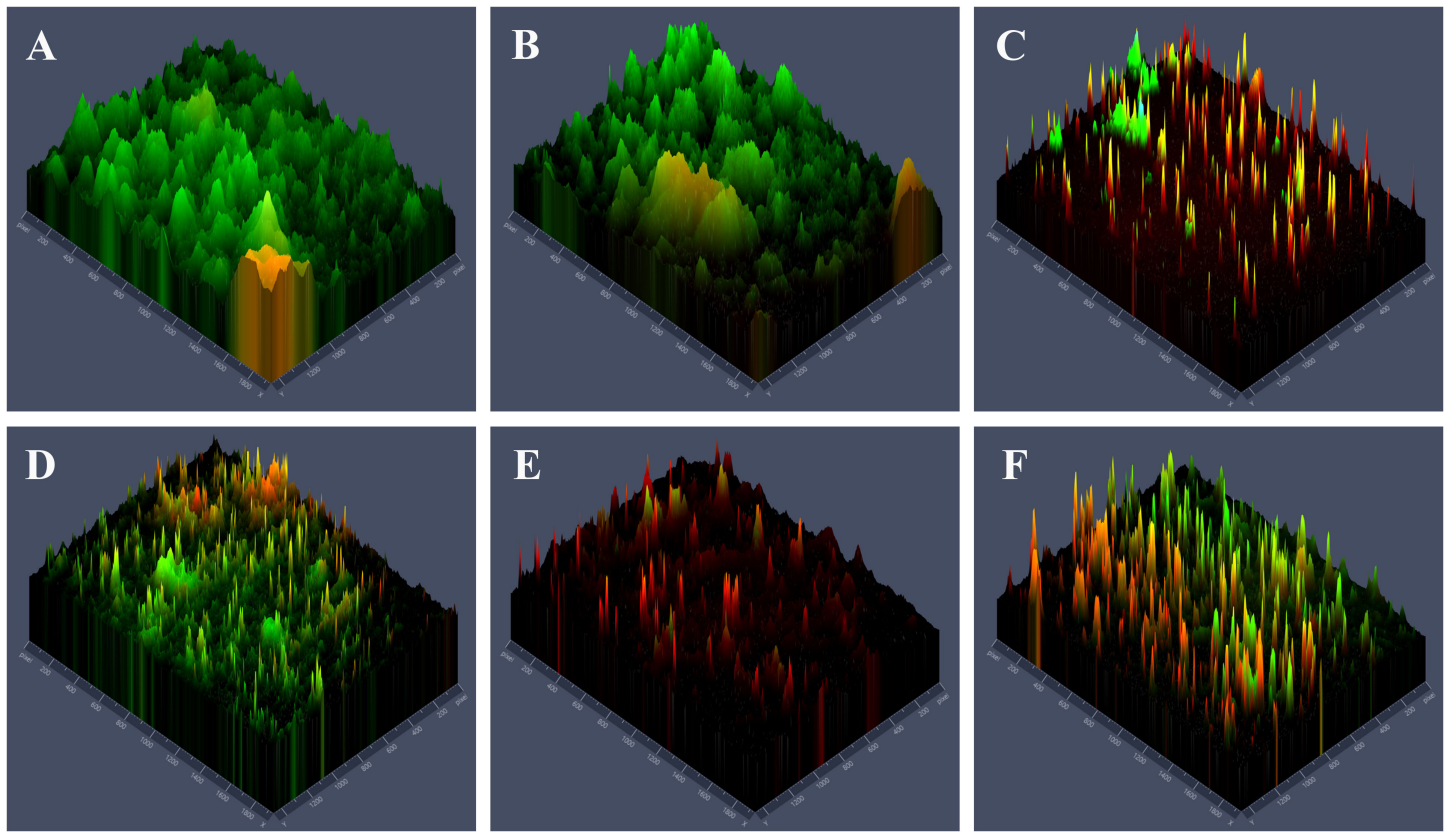
Fluorescence microscope observation of the effects of different treatments on the distribution of live and dead microbes and biofilm structure on the surface of dentin block, **(A)** PBS group, **(B)** TBO-aPDT-L group, **(C)** KI+TBO-aPDT-L group, **(D)** TBO-aPDT-H group, **(E)** KI+TBO-aPDT-H group, **(F)** 0.2% CHX group.

Live microbes were stained green which mainly in PBS group (**Figure 3A**) and TBO-aPDT-L group (**Figure 3B**), implicating these two treatments had no obvious effect on microbe viability. In the TBO-aPDT-H group and CHX group (**Figures 3D, F**), the proportion of red fluorescence increased, and the fluorescence distribution became sparse. Compared to aPDT or CHX treatments alone, a higher proportion of microbes stained with red fluorescence was observed in the KI+TBO-aPDT group (**Figures 3C, E**).

### 3.3 Phototoxicity of TBO-aPDT plus KI on hDPCs

**Figure 4:**
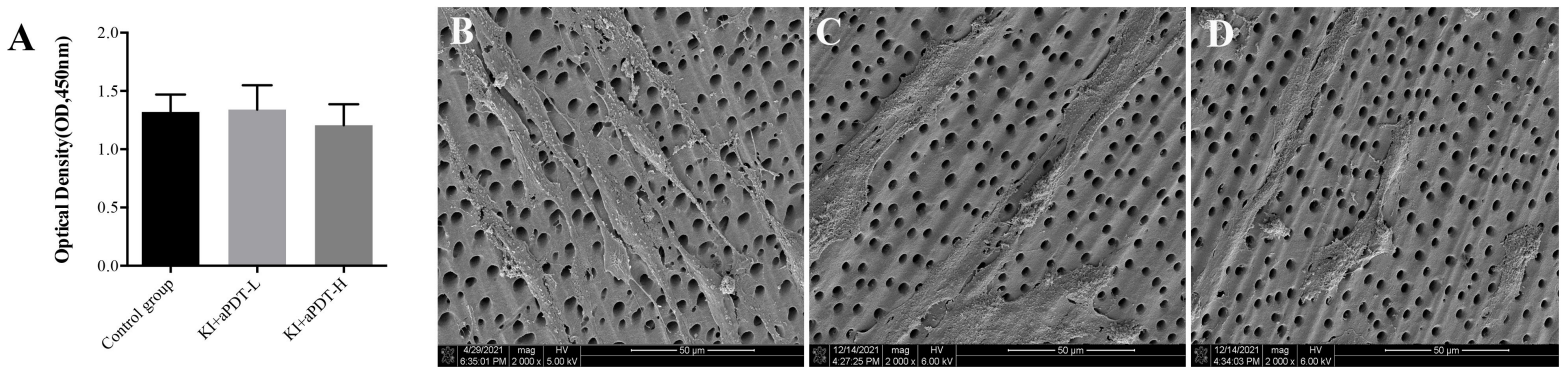
**(A)** CCK-8 analysis of hDPCs viability under the dentin block (mean ± SD, n=6/group). **(B)** SEM observation of hDPCs morphology under the dentin block from control group, magnification at 2000×, **(C)** SEM observation of hDPCs morphology under the dentin block from KI+TBO-aPDT-L group, magnification at 2000×, **(D)** SEM observation of hDPCs morphology under the dentin block from KI+TBO-aPDT-H group, magnification at 2000×.

As shown in **Figure 4A**, there is no significant difference regarding hDPCs viability between KI+TBO-PDT-treated groups and control group (*P*>0.05). According to SEM observation, hDPCs presented spindle shaped with elongated cytoplasmic processes in the control group (**Figure 4B**). However, KI plus TBO-aPDT treatment did not modify cell morphology of hDPCs beneath the dentin block in comparison with control group (**Figures 4C, D**).

### 3.4 Effect of TBO-aPDT plus KI on intercellular structures of microorganisms by TEM observation

**Figure 5:**
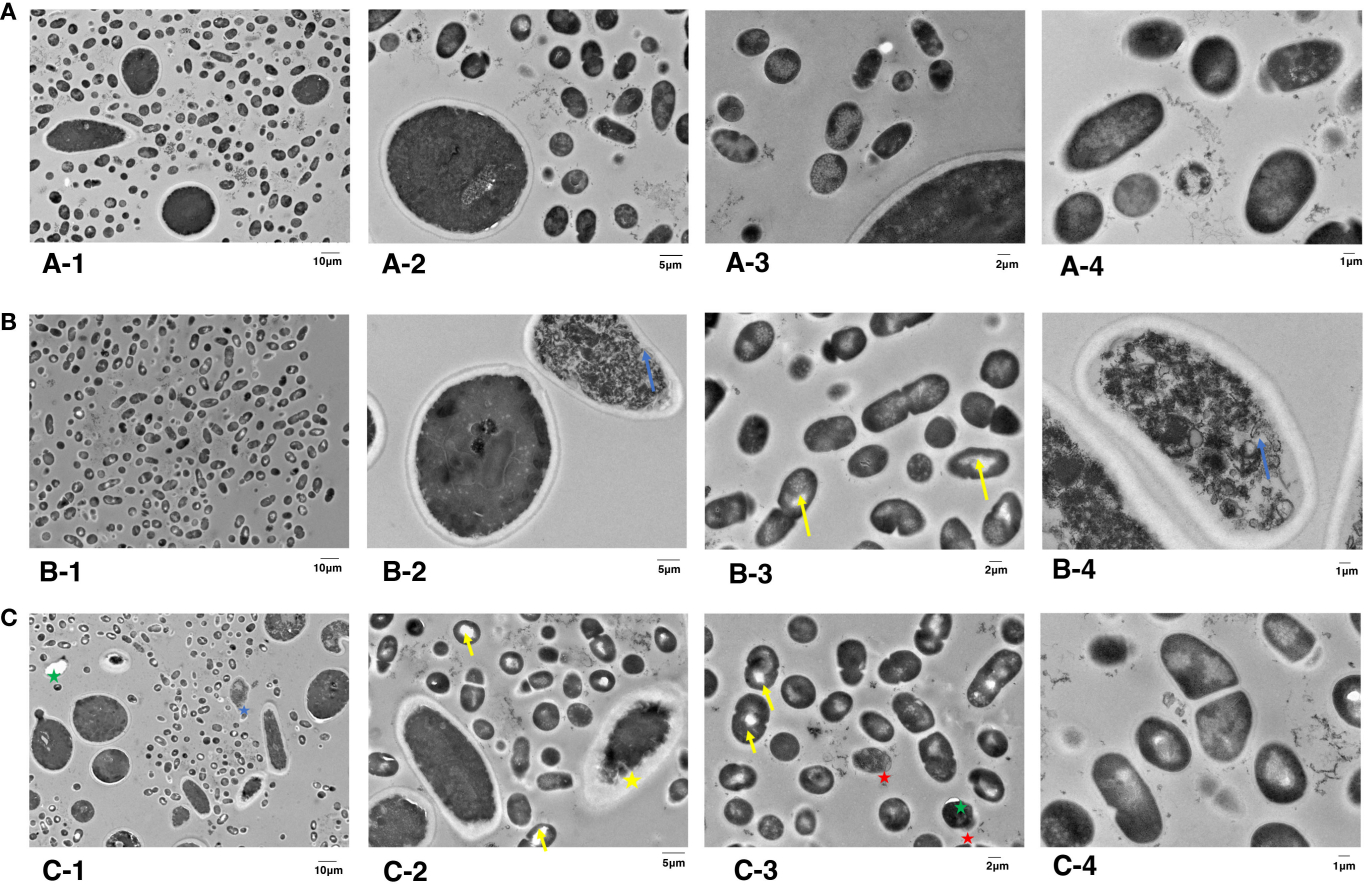
TEM images of subcellular structures of *S. mutans* and *C. albicans* on dentin blocks surface after different treatments. **(A)** Control group, magnified by 2,000× **(A1)**, 5,000× **(A2)**, 7,000× **(A3)**, and 12,000× **(A4)**. **(B)** KI+TBO-aPDT-L group, magnified by 2,000× **(B1)**, 5,000× **(B2)**, 7,000× **(B3)**, and 12,000× **(B4)**. **(C)** KI+TBO-aPDT-H group, magnified by 2,000× **(C1)**, 5,000× **(C2)**, 7,000× **(C3)**, and 12,000× **(C4)**.

Since there is no obvious change of cell morphology, we aimed to investigate whether there are the intracellular organelle damages inside the microbial cell by TEM. As shown in **Figure 5A**, two species of microorganisms in the control group were spherical-shaped with an intact structure, homogeneous electro-dense cytoplasm, and intact organelles. EPS matrix was randomly distributed among microorganisms. However, biofilm cells after treatment with aPDT plus KI demonstrated varying degrees of intracellular changes. After being treated with KI+TBO-aPDT-L, microbial cells underwent varying degrees of change in their cell structure (**Figure 5B**). *S. mutans* cells presented with regular spherical shape and clear cytoplasm structures. Light areas were observed in small number of *S. mutans* cells (yellow arrow). *C. albicans* cells have undamaged cell wall but presented severely cytoplasmic organelles (blue arrow, **Figure 5B-4**). The microbial cells experienced a greater number of intercellular changes after being treated with KI+TBO-aPDT-H (**Figure 5C**). Vacuole formation was observed not only in *C. albicans* but also in *S. mutans* (green star). The thickness between the cell membrane and cell wall of some *C. albicans* increases and condensed cytoplasm were observed (yellow star, **Figure 5C-2**). The cell membrane of *C. albicans* was damaged and lost its normal shape, in addition, cytoplasmic organelles were severely damaged (blue star, **Figure 5C-1**). The cell structure of *S. mutans* cells is abnormal, with partial detachment of cell walls and cytoplasmic membranes, and a large area of light transparent zone in the cell center (red star, **Figure 5C-3**).

### 3.5 Effect of TBO-aPDT plus KI on ROS production of microorganisms

**Figure 6:**
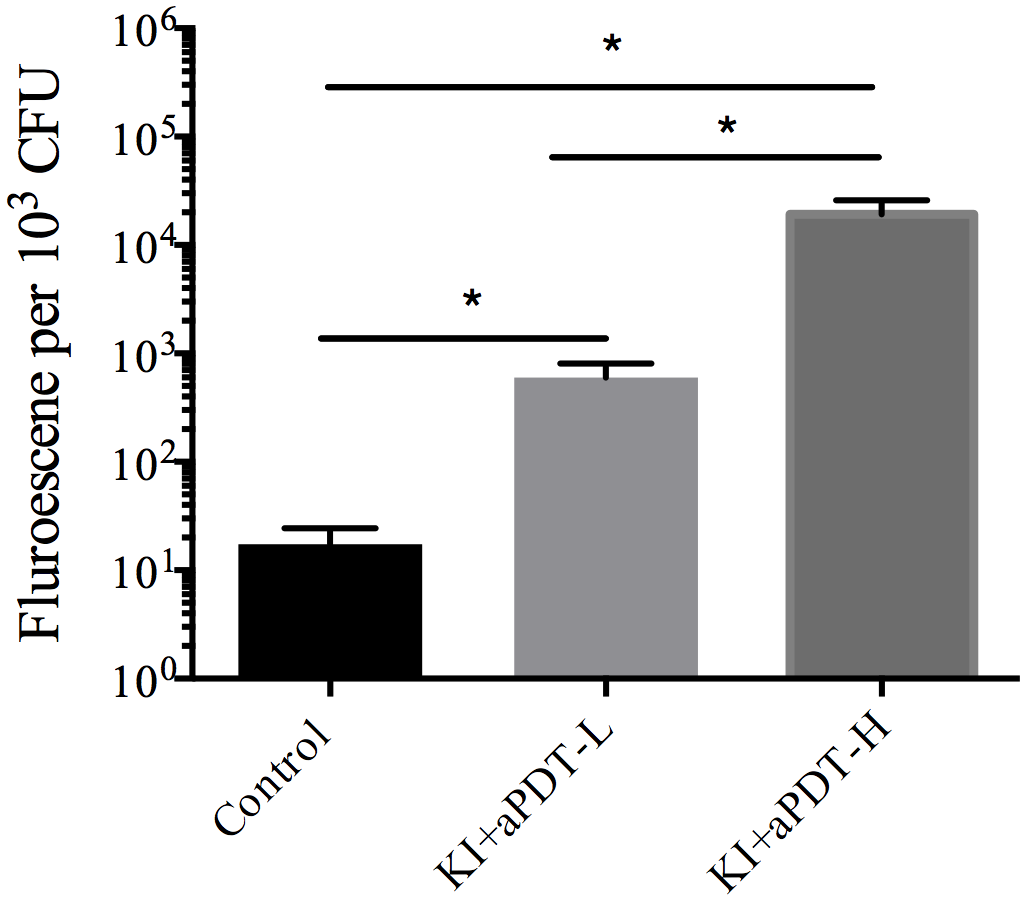
The effect on ROS level of microorganisms in mixed-species biofilm after treating with TBO-aPDT and KI (mean ± SD, n=6/group). *P < 0.05.

We then assessed whether KI plus TBO-aPDT induces ROS generation inside the cell, which has been proposed among the possible mechanism of light irradiation. Compared to control group, KI+TBO-aPDT treatment induced ROS generation in microbes (*P*<0.05). The most pronounced ROS yield was detected when mixed biofilm cells treated with KI+TBO-aPDT-H (**Figure 6**).

### 3.6 Effect of TBO-aPDT plus KI on cell membrane permeability, cell membrane potential and apoptosis of microorganisms

**Figure 7:**
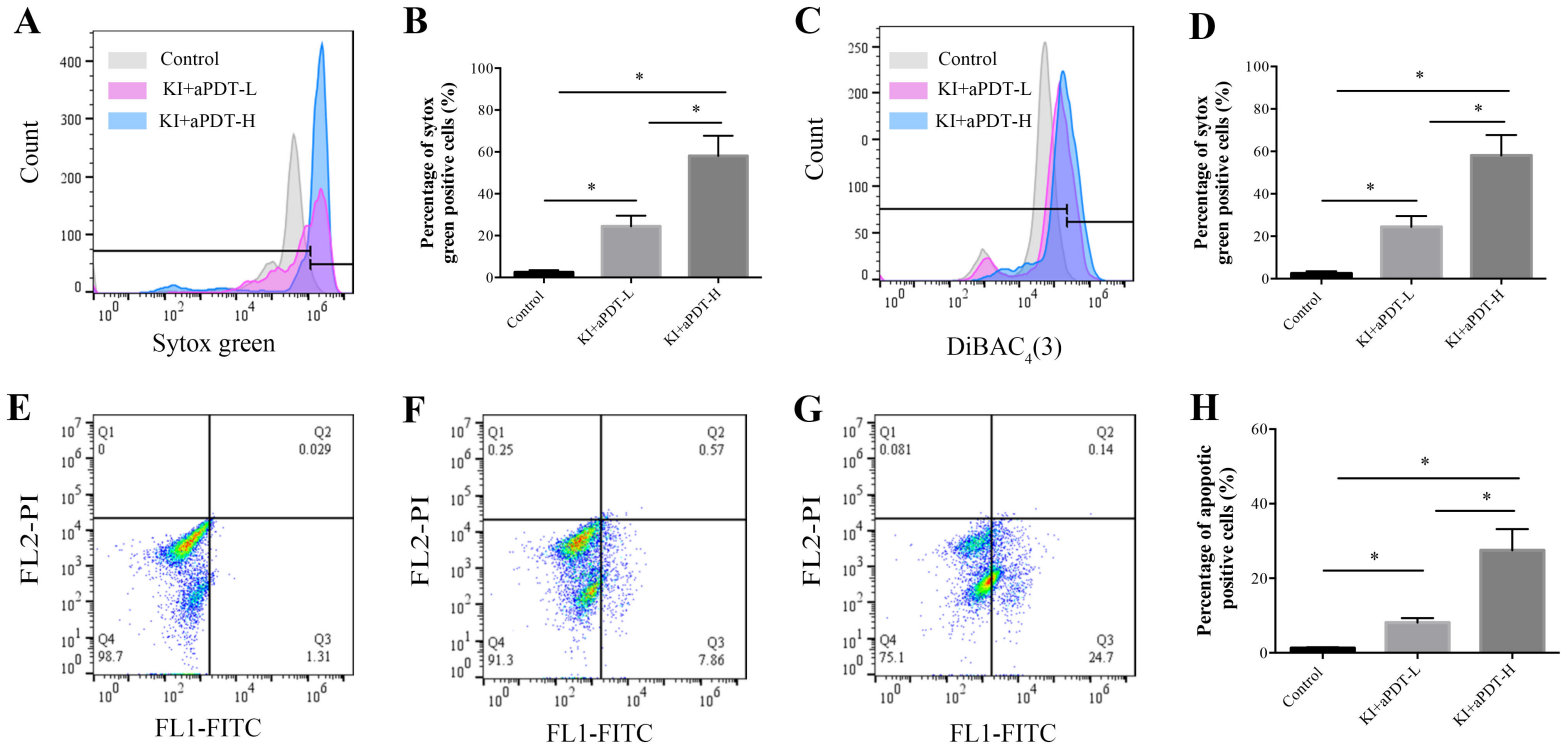
Flow cytometry analysis of cell membrane permeabilization, cell membrane potential, and cell apoptosis of microbial cells after different treatments. **(A)** Histogram of SYTOX green staining. **(B)** The proportion of microbial cell membrane permeabilization after different treatments (mean ± SD, n=6/group). **(C)** Histogram of DiBAC_4_(3) staining. **(D)** The proportion of microbial cell membrane depolarization after different treatments (mean ± SD, n=6/group). Detection of cell apoptosis in microorganisms after different treatments by staining with Annexin V-FITC/PI using flow cytometry **(E-H)**. **(E)** Scatter plot of control group. **(F)** Scatter plot of KI+TBO-aPDT-L group. **(G)** Scatter plot of KI+TBO-aPDT-H group. **(H)** The proportion of microorganisms undergoing apoptosis after treatment in different groups (mean ± SD, n=6/group). *P < 0.05.

SYTOX green could easily penetrate compromised plasma membranes and bind to nucleic acid and then emit green fluorescence. The percent of fluorescent cells in mixed-species biofilm treated with KI+TBO-aPDT is significantly increased compared to the control group, indicating that KI+TBO-aPDT treatment induced rapid permeabilization of cell membrane (**Figure 7A**). Approximately 29.35% and 58.07% of microbial cells showed high SYTOX green fluorescence in KI+TBO-aPDT-L group and KI+TBO-aPDT-H group, respectively (**Figure 7B**).

DiBAC_4_(3) has ability to enter depolarized cells and bind to hydrophobic core of the lipid membrane, emitting fluoresces. As shown in **Figure 7C**, the percent of fluorescent cells in mixed biofilms treated with KI+TBO-aPDT is higher than the control group. Approximately 17.52% and 35.2% of microbial cells showed high DiBAC_4_(3) fluorescence in KI+TBO-aPDT-L group and KI+TBO-aPDT-H group, respectively (**Figure 7D**).

The Annexin V-FITC apoptosis kit was used to detect the displaced phosphatidylserine on cell membrane. As demonstrated in **Figures 7E–G**, cells were sorted into necrotic (Q1, annexin V negative and PI negative), late apoptotic cells (Q2, annexin V positive and PI positive), early apoptotic (Q3, annexin V positive and PI negative), and living (Q4, annexin V positive and PI negative). Compared to control group, KI+TBO-aPDT-L treatment induced early apoptosis and identified approximately 8.12% of early apoptotic cells. KI+TBO-aPDT-H treatment showed more increase in early apoptotic cells with a percentage of 27.5%. However, KI+TBO-aPDT treatment did not trigger late apoptosis of microbial cells in biofilm (**Figure 7H**).

### 3.7 Effect of TBO-aPDT plus KI on gene expression of microorganisms

**Figure 8:**
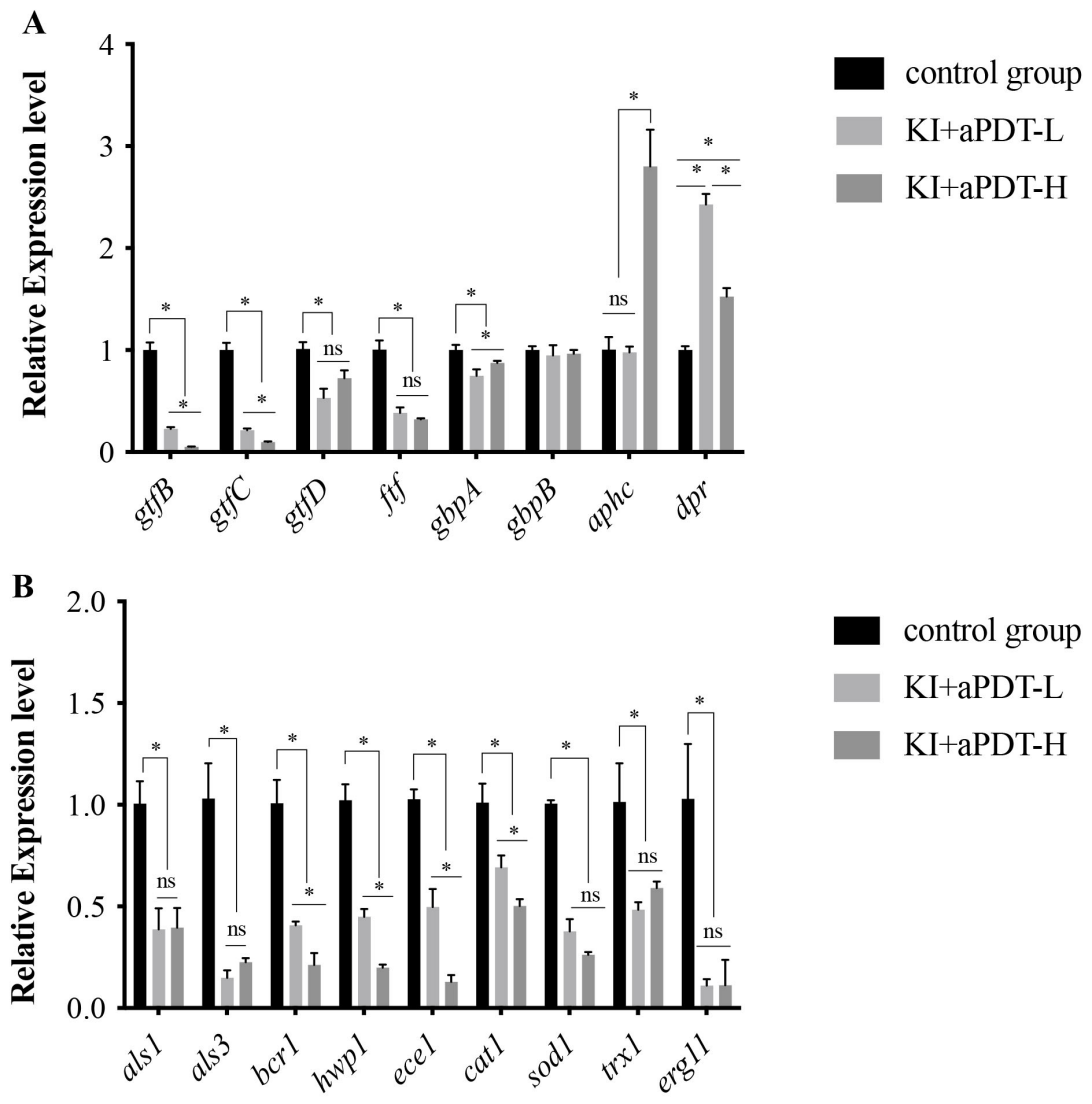
RT-PCR analysis of *S. mutans* and *C*. *albicans* specific genes in mixed-species biofilm after treating with TBO-aPDT plus KI. **(A)** *S. mutans* genes. **(B)** *C*. *albicans* genes. *P < 0.05; ns, no significance.

To evaluate the mechanisms of TBO-aPDT plus KI on mixed-species biofilm, we analyzed changes in *S. mutans and C. albicans* gene expression levels when exposure to KI+TBO-aPDT treatment. We evaluated bacteria genes which are essential in biofilm development including those related to glucan formation (*gtfB, gtfC, gtfD and ftf*), adhesion (*gbpA and gbpB*), and oxidative stress tolerance (*aphcF and dpr*). Briefly, KI+TBO-aPDT-L treatment decreased mRNA transcription of *gtfB* by 4.4-fold, *gtfC* by 4.7-fold, *gtfD* by 1.9-fold, *ftf by* 2.6-fold, and *gbpA* by 1.3-fold as compared to untreated control group (**Figure 8A**). Meanwhile, KI+TBO-aPDT-L treatment increased *dpr* gene expression by 2.4-fold in comparison with untreated control group. KI+TBO-aPDT-H treatment decreased mRNA transcription of *gtfB* by 18.8-fold, *gtfC* by 10.1-fold, *gtfD* by 1.4-fold, *ftf by* 3.1-fold, and *gbpA* by 1.1-fold as compared to untreated control group (**Figure 8A**). KI+TBO-aPDT-L treatment increased *aphcF and dpr* gene expression by 2.8-fold and 1.5-fold respectively when compared to untreated control group (**Figure 8A**).

We also investigated fungal genes which are associated with adhesion (*als1, als3, hwp1*), biofilm formation (*bcr1, ece1*), oxidative stress tolerance (*cat1, sod1, trx1*), and cell membrane function (*erg11*). Briefly, KI+TBO-aPDT-L treatment decreased mRNA transcription of *als1* by 2.6-fold, *als3* by 6.7-fold, *hwp1* by 2.2-fold, *bcr1* by 2.5-fold, *ece1* by 2.0-fold, *cat1* by 1.4-fold, *sod1* by 2.6-fold, *trx1* by 1.3-fold and *erg11* by 9.0-fold as compared to untreated control group (**Figure 8B**). KI+TBO-aPDT-H treatment decreased mRNA transcription of *als1* by 2.5-fold, *als3* by 4.4-fold, *hwp1* by 5.0-fold, *bcr1* by 4.7-fold, *ece1* by 7.7-fold, *cat1* by 2.0-fold, *sod1* by 3.8-fold, *trx1* by 1.7-fold and *erg11* by 8.9-fold when compared to untreated control group (**Figure 8B**).

## 4 Discussion

The synergistic cooperation between *S. mutans* and *C. albicans* has been extensively studied in the context of caries. Given the aggressive damage caused by *S. mutans* and *C. albicans* that develop hard-to-remove and highly drug-resistant biofilms, it is imperative to develop cost-effective and safe therapies to disrupt mature biofilms or prevent their formation. KI is a non-toxic salt and is an approved drug for antifungal therapy. The combination of KI into aPDT procedure could give rise to several benefits, including improved antimicrobial efficacy, reduced photosensitizer concentration and irradiation time, and higher tolerance to antioxidant activities. This study provides significant evidence of the anti-biofilm effect of KI in combination with TBO-aPDT against the carious biofilm formed by *S. mutans* and *C. albicans*. Although a few papers have in part evaluated some aspects of KI in combination with aPDT, this is the first study to investigate the antimicrobial effect of KI in combination with aPDT on mixed-species biofilm and shed light on the antimicrobial mechanisms.

There are many *in vitro* caries biofilm models described in previous literature (Cavalcanti et al., 2014; Maske et al., 2017). Many reports have used synthetic surfaces such as glass, polystyrene, or hydroxyapatite to grow biofilm *in vitro*. However, these surfaces cannot mimic the fine details of dentine microanatomy or reproduce clinically relevant biofilm formation. In addition, *in vitro* biofilm models used for evaluating therapeutic interventions often use single strain, which overlooks the physiological interactions among microbes in the oral cavity. In light of these limitations, the highly structured cross-kingdom biofilm was constructed on vertically placed dentin blocks in the present study to mimic the clinical situation as much as possible.

Here, we adopted MTT assay, CFU assay, and crystal violet staining to evaluate the antimicrobial ability of TBO-aPDT plus KI against cross-kingdom biofilm. For better comparison, we included aPDT and CHX treatment as controls. The survival counts and metabolic activity assessments noted that aPDT alone has unsatisfactory effect on mixed-species biofilm formed by *S. mutans* and *C. albicans*. Quishida et al. observed that curcumin-mediated aPDT could inhibit multi-species biofilm but only led to 1-2 logs in microbial counts (Quishida et al., 2016). Teixeira and his co-workers found that the bacterial reduction of saliva-derived biofilm subject to methylene blue-mediated aPDT is lower than 1 log (Teixeira et al., 2012). These results were in line with our finding, indicating that aPDT has a limited effect on multispecies biofilm. Interestingly, TBO-aPDT plus KI exhibited a significant effect on microbial counts, metabolic activity, and biofilm biomass reduction of mixed-species biofilm, whose antibiofilm effect is greater than aPDT and CHX. Biofilm dispersion ability is extremely significant for a new antimicrobial protocol since the residual biofilm matrix serves as a nutrient source for residual viable bacteria, enabling their continued proliferation and contributing to the recurrence of infections. According to the USA Food and Drug Administration’s Tentative Final Monograph for Healthcare Antiseptic Drug Products, a reduction of at least 3 log steps must be achieved to state a bactericide effect of a specific treatment (Boyce and Pittet., 2002). The quantitative CFU assay suggested KI+TBO-aPDT-H group could trigger more than 3 logs in microbial reduction, which indicates KI in combination with aPDT could exert a bactericide effect. The dramatic antibiofilm ability of TBO-aPDT plus KI is mainly attributed to the generation of iodine molecules and hydrogen peroxide, which was proved in our previous report (Li et al., 2022). And, the reason responsible for the difference on antibiofilm capacity among three treatments is mainly due to their antimicrobial mechanism. It is known that ROS generated by aPDT procedure has a limited diffusion distance and short lifetime, and thus has a limited effect on biofilm with abundant matrix (Alves et al., 2014). CHX is a bisbiguanide antiseptic that could interact with lipopolysaccharides or lipid of cell membrane and then change the permeability of bacterial cell membranes to kill bacteria (Varoni et al., 2012). However, the antimicrobial effect of CHX is confined by rich matrix surrounded by multi-species biofilm since CHX with cationic charge would preferentially binds to negatively charged extracellular matrix (Souza et al., 2019). In contrast to aPDT or CHX, molecular iodine and hydrogen peroxide generated by KI+TBO-aPDT process are synergistic and sustained killing (Zubko and Zubko, 2013). Iodine as a small molecule, could penetrate into microbes quickly and oxidize essential proteins, nucleotides and fatty acids, eventually leading to cell death. Hydrogen peroxide rapidly penetrates into cell membrane of microorganisms, participate in the Fenton reaction to generate free hydroxyl radicals, and consequently cause irreversible damage to cells.

Despite a few reports regarding aPDT in combination with KI in the elimination of biofilm, there is no evidence elucidating the mechanisms involved in the combination strategy of aPDT plus KI. This is the first study to shed light on the antimicrobial mechanisms of KI in combination with TBO-aPDT. Earlier in 2007, Kohanski et al. proposed different types of drugs could kill bacteria via the activation of electron transfer in the respiratory chain and the induction of massive ROS accumulation (Kohanski et al., 2007). Hence, we firstly checked whether TBO-aPDT plus KI could yield ROS burden in mixed-species biofilm or not. Our results showed the ROS level inside mixed-species biofilm after treatments was markedly increased. The increase in endogenous ROS level is related to TBO concentration and light dose.

TEM observation revealed the disorganization and dissolution of intracellular organelles in microbes after treatments, while the integrity of the cell wall remained intact. It is reasonable to assume that the cell membrane properties change of two microbes may occur after KI+TBO-aPDT treatment and trigger the penetration of iodine and hydrogen peroxide to injure cell organelles. The reactive ROS would directly react with poly-unsaturated fatty acids on the cell membrane, cause lipid peroxidation and further alter cell membrane properties, thereby destroying cell membrane function. Cell membrane is the essential biological barrier of microorganisms that play a role in nutrient and information exchange, and also a target for many antimicrobial agents. According to ROS measurement and TEM observation, we used flow cytometry to assess cell membrane damage in biofilm cells after treatments. The flow cytometry analysis revealed KI+TBO-aPDT treatment induce cell membrane permeabilization and depolarization in *S. mutans* and *C. albicans*. Recently, apoptosis-like death in fungus or bacteria was also observed and it involved several important traits such as ROS accumulation, cell membrane depolarization, cell membrane permeabilization and DNA fragmentation (Wang et al., 2015; Kim and Lee, 2020). In the view of the obtained results, it can be reasonably speculated that KI plus aPDT treatment may induce apoptosis in microbial cells. Consistent with our speculation, FITC-Annexin V staining showed PS externalization was evident in *S. mutans* and *C. albicans* cells after KI plus aPDT treatment.

RT-PCR was further performed to explore the antibiofilm mechanisms of KI in combination with TBO-aPDT at the genetic level. We measured the expression levels of *S. mutans* genes that are associated with glucan synthesis, initial adherence, and oxidative stress tolerance. When treated with KI+TBO-aPDT, the expression of *gtfBCD* of *S. mutans* in mixed-species biofilm were significantly downregulated. As known, gtfs secreted by *S. mutans* especially *gtfB* contributes to the production of an extensive extracellular matrix in the presence of *C. albicans*, leading to virulent mixed biofilms under a sugar-rich environment (Ellepola et al., 2017). The expression inhibition of these genes following KI+TBO-aPDT treatment may weaken the synergistic relationship between *S. mutans* and *C. albicans* and reduce cross-kingdom biofilm formation and pathogenicity. GBPs have been proven to be important for mediating bacterial binding to the glucan produced by GtfB adsorbed onto *Candida* surface (Fujita et al., 2007). GbpA has a relationship with GTF production and may regulate the sucrose-dependent adhesion of *S. mutans* (Matsumoto-Nakano et al., 2007). The downregulation of *gbpA* may influence the co-adhesion between *S. mutans* and *C. albicans*, resulting in sparse co-species biofilm formation. Meanwhile, we also evaluated the transcriptional level modulations of adhesin and biofilm formation genes in *C. albicans* after exposure KI+TBO-aPDT treatment. The agglutinin-like sequence family is located in the cell wall of *C. albicans* and is the key to *C. albicans* in the interaction with other bacteria during biofilm development (Klotz et al., 2007). A recent study has used *C. albicans* homozygous knockout strains including *△△als1*/*△△als3* to investigate the role of adhesins in the interkingdom colonization between *S. mutans* and *C. albicans* (Ren et al., 2022). The results suggested that the *als1*/*als3* deletion resulted in a severe reduction of coassembly between *C. albicans* and *S. mutans*, indicating that *C. albicans* could directly bind to *S. mutans* through these surface proteins. *Hwp1* is the first cell surface protein known to be required for *C. albican*s biofilm formation *in vivo* and is thus an excellent therapeutic target (Nobile et al., 2006). *Bcr1* is required for cell-substrate adherence and biofilm formation in *C. albicans*, which could regulate the expression of downstream target genes such as *als1*, als3, and *hwp1* (Finkel et al., 2012). Downregulation of these genes implied that KI in combination with aPDT may impair, at least in part, some crucial virulence factors of *C. albicans* and reduce *C. albicans’s* binding to *S. mutans* as well as subsequent biofilm formation.

In the present study, we observed notable ROS accumulation in mix-species biofilm after KI+ TBO-aPDT treatment, therefore we measured the transcription levels of oxidative stress tolerance gene. Upregulation of oxidative stress tolerance genes in *S. mutans* is fairly predictable after treatments since DCFH-DA assay indicated that the increase endogenous ROS levels in mixed-species biofilm cells. The increased expression levels of *aphcF* and *dpr* suggested that their involvement in protection against oxidative stress produced by KI+TBO-aPDT treatment. AphC F and Dpr are essential proteins that involved in oxidative stress tolerance in *S. mutans*. Dpr could suppress the Fenton reaction pathway which mediates the generation of hydroxyl radicals via capturing free Fe (Fujishima et al., 2013). Remarkably, however, the transcription levels of oxidative stress genes in *C. albicans* were dramatically downregulated after exposure to KI+TBO-aPDT treatment, suggesting that KI+TBO-aPDT treatment may impair the protective system against oxidative stress. This is line with a previous report that demonstrated downregulation of oxidative stress genes in *C. albicans* after 5-octylidenethiazolidine- 2,4-dione treatment (Feldman et al., 2016). KI+TBO-aPDT treatment attacks microbes from multiple angles, therefore, the treatment is likely to be more effective in eradicating a broader range of microorganisms involved in dental caries. Besides, it would be difficult for microorganisms develop resistance towards the treatment. Furthermore, by attacking bacteria from multiple angles simultaneously, KI+TBO-aPDT may be able to achieve faster results compared to single-target antimicrobials. However, the clinical data of KI+TBO-aPDT treatment is blank and more work should be done to determine its clinical efficacy and safety profile.

## 5 Conclusion

In conclusion, our results demonstrate KI potentiates TBO- aPDT against cross-kingdom biofilm from dentin blocks without affecting hDPCs viability. The killing mechanisms of KI in combination with aPDT are mainly due to increased ROS levels, impaired cell membrane function, cell apoptosis and modulation in gene expression level of microorganisms. This study suggests that KI plus TBO-aPDT may represent a potential approach in caries treatment as well as in other cross-kingdom biofilm-associated diseases.

## Data Availability

The raw data supporting the conclusions of this article will be made available by the authors, without undue reservation.
