# Supplementary material for: Deciphering the killing mechanisms of potassium iodide in combination with antimicrobial photodynamic therapy against cross-kingdom biofilm
Source: Front Cell Infect Microbiol. 2024 Oct 15;14:1444764. doi: 10.3389/fcimb.2024.1444764 (PMC11518841; doi:10.3389/fcimb.2024.1444764)
Supplement: Supplementary file 1 [file Table1.docx]

Supplementary Material

Table S1. Primer sequences of *S. mutans* used in RT-qPCR.

| Primer | Sequence |
| --- | --- |
| *gtfB*-F | 5’-TGCCGCAGTCCCTTCTTATTC-3’ |
| *gtfB*-R | 5’-GCCATGTATTGCCCGTCATCT-3’ |
| *gtfC*-F | 5’-GTGCGCTACACCAATGACAGAG-3’ |
| *gtfC*-R | 5’-GCCTACTGGAACCCAAACACCTA-3’ |
| *gtfD*-F | 5’-TACCTTGGGCACCACAACACT-3’ |
| *gtfD*-R | 5’-TGCCGCCTTATCATCCTCACT-3’ |
| *ftf*-F | 5’-AAATATGAAGGCGGCTACAACG-3’ |
| *ftf*-R | 5’-CTTCACCAGTCTTAGCATCCTGAA-3’ |
| *gbpA-*F | 5’-GCCGAGCGTATCAGTACAGTTGAG-3’ |
| *gbpA-*R | 5’-CCGTCATCAGGCACAGAACCAC-3’ |
| *gbpB-*F | 5’-CAGCAGCGGCAGGATATAGAGTTG-3’ |
| *gbpB-*R | 5’-ACGTGTCCATAACCGCCATCATTC-3’ |
| *aphc*-F | 5’-GGGATCCTTCTCATGTTCTCTC-3’ |
| *aphc*-R | 5’-CATCTGCATTGACTTCCATCATT-3’ |
| *dpr-*F | 5’-ACCAAGGCTGTACTTAATCAGG-3’ |
| *dpr-*R | 5’-AGGATGCAGATAAAGGAAGCC-3’ |
| *16S rRNA*-F | 5’-AGCGTTGTCCGGATTTATTG-3’ |
| *16S rRNA*-R | 5’-CTACGCATTTCACCGCTACA-3’ |

Table S2. Primer sequences of *C. albicans* used in RT-qPCR.

| Primer | Sequence |
| --- | --- |
| *als1*-F | 5’- AGCTGTTGCCAGTGCTTC -3’ |
| *als1*-R | 5’- AATGTGTTGGAAGGTGAG -3’ |
| *als3*-F | 5’- CTAATGCTGCTACGTATAAT -3’ |
| *als3*-R | 5’-TGTATCTCCCGGACTTGCACT-3’ |
| *bcr1*-F | 5’- CTTCAGCAGCTTCATTAACACCTA -3’ |
| *bcr1*-R | 5’- TCTTGGATCAGGTGTACTTTTCAA -3’ |
| *cat1*-F | 5’- ATTTCATCCACACCCAAAAGAGA -3’ |
| *cat1*-R | 5’- TTGCTAGTCAAGTAATCCCAAAACA -3’ |
| *ece1*-F | 5’- GCTGGTATCATTGCTGATAT -3’ |
| *ece1*-R | 5’- TTCGATGGATTGTTGAACAC -3’ |
| *erg11*-F | 5’- AAGAATCCCTGAAACCAA -3’ |
| *erg11*-R | 5’- CAGCAGCAGTATCCCATC -3’ |
| *hwp1*-F | 5’- TGGTGCTATTACTATTCCGG -3’ |
| *hwp1*-R | 5’- CAATAATAGCAGCACCGAAG -3’ |
| *sod1*-F | 5’- TTGAACAAGAATCCGAATCC -3’ |
| *sod1*-R | 5’- AGCCAATGACACCACAAGCAG -3’ |
| *trx1-*F | 5’- TCAATTGGGTTCTTTAGCACA -3’ |
| *trx1-*R | 5’- TGACACGATTGACTTCTTCACC -3’ |
| *18S rRNA*-F | 5’-CTACGCATTTCACCGCTACA-3’ |
| *18S rRNA*-R | 5’-CTACGCATTTCACCGCTACA-3’ |
