# Supplementary figures and images for: Deciphering the killing mechanisms of potassium iodide in combination with antimicrobial photodynamic therapy against cross-kingdom biofilm
Source: Front Cell Infect Microbiol. 2024 Oct 15;14:1444764. doi: 10.3389/fcimb.2024.1444764 (PMC11518841; doi:10.3389/fcimb.2024.1444764)

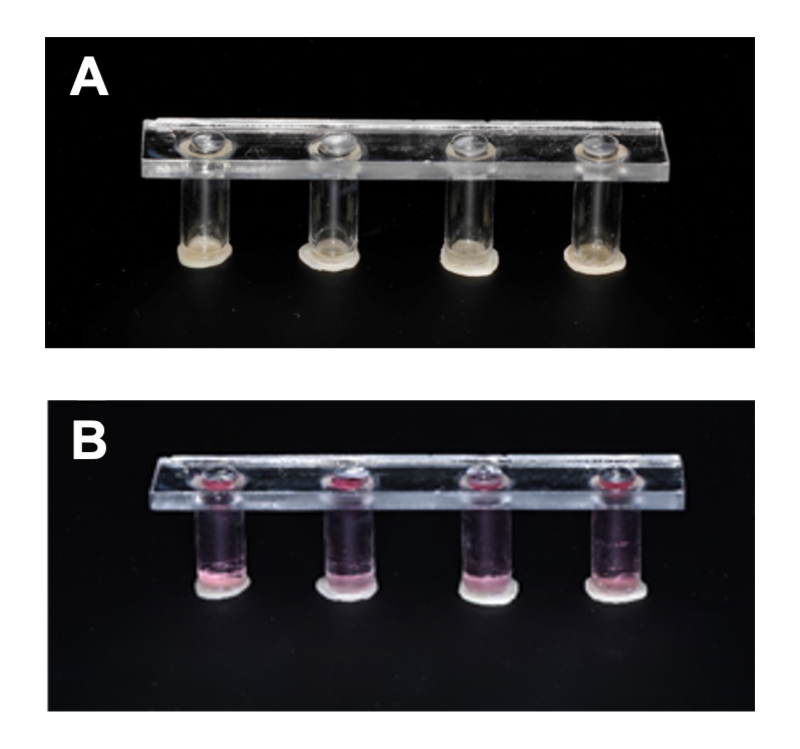

Supplement: Supplementary file 2 [file Image1.tiff]
